# Exposure of *Pisum* *sativum* L. Seeds to Methomyl and Imidacloprid Cause Genotoxic Effects in Pollen-Mother Cells

**DOI:** 10.3390/biology11111549

**Published:** 2022-10-23

**Authors:** Sazada Siddiqui, Sulaiman A. Alrumman

**Affiliations:** Department of Biology, College of Science, King Khalid University, Abha 61413, Saudi Arabia

**Keywords:** insecticides, methomyl, imbraclaobrid, cytotoxicity, genotoxicity, *Pisum sativum* L.

## Abstract

**Simple Summary:**

Pesticides are highly effective and widely accepted for safeguarding crops from pests, thus substantially enhancing agricultural productivity. In relation to the ecological toxicity caused by pesticides and their potential impact on nontarget entities, the purpose of this investigation was to assess the action of insecticides methomyl (ME), and imidacloprid (IM) on *Pisum sativum* L. The results indicate that these insecticides decrease the mitotic index (MI) and micronucleus frequency (MNF) in a dose-dependent manner. In addition, they exhibited a substantial genotoxic effect on *P. sativum*. Further studies to assess the impact of these insecticides on nontarget host plants should be explored at the molecular and biochemical levels in order to determine the mechanism of action of these insecticides.

**Abstract:**

Pesticides are commonly used in modern agricultural systems to protect the plants from pests. Even though they potentially increase the crop yield, they have undesirable toxic effects on the consumers of plant products and nontarget host plants. However, there are limited studies to demonstrate the cytological changes induced by pesticides on plant cells. In the present study, we assess the cytological changes induced by two most commonly used insecticides, methomyl (ME) and imidacloprid (IM), using *Pisum sativum* L. as model plant system. *P*. *sativum* seeds were exposed to various concentrations of ME and IM (0.1, 0.2, 0.3, 0.4 and 0.5%) for 1, 3, and 6 h, and their effects on seed germination (SG), radicle length (RL), mitotic index (MI), chromosomal aberrations frequency (CAF), and micronucleus frequency (MNF) were studied. The results indicate that these insecticides decrease MI in root-tip cells, and increase in the MNF in pollen-mother cells in a dose-dependent manner. Additionally, insecticide-treated groups showed a dose- and time-dependent increase in the percentage of aberrant meiotic cells. Clumped nuclei (CNU), stickiness (STC), bridges (BRs), laggards (LGs), secondary association (SA), and precocious separation (PS) were among the frequently observed anomalies. The findings of this study indicate that commonly used insecticides ME and IM have substantial genotoxic effects on the root-tip and pollen-mother cells of *P. sativum* L.

## 1. Introduction

Pesticide use is a highly effective and widely accepted method of safeguarding crops from pests, and it substantially enhance agricultural productivity [1,2]. However, since pesticides are widely used and have a biocidal effect, they may have detrimental effects on nontarget entities such as plants, mammals, aquatic biota, and soil microbes [3]. The insecticide imidacloprid (IM) is a member of the neonicotinoid chemical group that is commonly used globally for agricultural purposes. It has great displacement capability, allowing for it to enter groundwater [4,5]. It functions as an antagonist of the nicotinic acetylcholine receptor in the central nervous systems of insects, activating neurons, generating fatigue, and interfering in nerve impulse transmission [6,7]. Methomyl (ME) belongs to the monomethyl carbamate group of insecticides used globally for controlling pests on a variety of food crops, tobacco, and cotton [8,9,10,11]. It is used to protect crops due to its highly efficient biological action [12,13].

Various plant species (*Arabidopsis thaliana, Allium cepa, Arabidopsis thaliana, Vicia faba, Hordeum vulgare*, and *Tradescantia* sp.) have been used for evaluating the detrimental effects of these substances as they are immediate biologic receivers of pesticides [14]. Even though these compounds are intended to have inhibitory effects on plant growth, few studies have revealed that plants can be an effective material for assessing the genotoxicity caused by the action of herbicides [15,16]. A few studies reported the genotoxic nature of commercial preparations of methomyl [17,18,19,20]. DNA damage is an initial biological phenomenon that has the potential to disrupt biological structures and processes, and cause genotoxic syndromes linked to carcinogenic processes [21,22,23]. According to recent analysis, a wide range of species experience carcinogenic developments for a variety of reasons, including DNA damage caused by chemical pollutants [24]. In addition, the fitness and reproductive success of natural biota could be affected by unrepaired or incorrectly repaired DNA damage in germ cells, which would eventually result in a long-term decline in the quality of the ecosystem [22,25]. In order to understand the toxicity caused by insecticides on nontarget entities such as plants, the present investigation assesses the action of the most widely used insecticides, ME and IM, on the mitotic and meiotic cells of plants using *P. sativum* L. as an experimental model.

## 2. Materials and Methods

### 2.1. Procurement of Seeds and Chemicals

*Pisum sativum* L. seeds were obtained from a certified dealer at a local market in Abha, Aseer region, Kingdom of Saudi Arabia. Insecticides methomyl (CAS No. 16752-77-5) and imidacloprid (CAS No. 138261-41-3) were purchased from Sigma Chemical Corporation, Saint Louis, United States).

### 2.2. Seed Treatment and Sowing

The stock solution of insecticides was prepared by dissolving 5 g of ME and IM each in 500 mL of distilled water. Further 0.1 to 0.5% concentrations of ME and IM were prepared from a stock solution. *P. sativum* seeds were soaked in distilled water for 12 h, followed by treatment with different concentrations of ME and IM (0.1%, 0.2%, 0.3%, 0.4%, and 0.5%) for 1, 3, and 6 h with recurrent shaking in a mechanical shaker. The seeds were rinsed under running tap water for 10 min to completely remove the insecticides that were stuck to the seed coat. A set of seeds that were given the same treatment as the experimental seeds but devoid of insecticide treatments were used as control seeds for comparison. Six sets of treated seeds and control seeds were individually sown using a complete randomized block design (CRBD) with three replicates from October to December (2020–2021). There were 300 seeds in each treatment group; 100 seeds were sown with a seed-to-seed distance of 25 cm and a row-to-row distance of 40 cm in each 6 × 6 m plot. In the treatment groups, fertilizers were not used.

### 2.3. Determination of Seed Germination (SG) and Radicle Length (RL)

In each Petri plate covered with a two layers of filter paper, 30 seeds presoaked with water were placed with adequate spacing and soaked in 10 mL of the insecticide solution with various concentrations (0.1 to 0.5%). The experiments were carried out in a growth chamber and maintained under dark conditions (for 4 h) for seed germination. After that, a 16/8 h light/dark photoperiod with light intensity of 500 l mol/m^2^/s was established. During the experiment, temperature of 30 °C and humidity of 60% were maintained. The seeds exposed to insecticides for 1, 3, and 6 h were monitored for seed germination for three days. Seed germination was determined by observing radicle formation. A millimeter ruler was used to measure the length of the newly formed roots at every 24 h interval. The entire experiment was repeated thrice under similar conditions.

$$\mathrm{Seed}\;\mathrm{gerimation}\;\left(\%\right)=\frac{\mathrm{No}.\;\mathrm{of}\;\mathrm{seeds}\;\mathrm{germinated}}{\mathrm{Total}\;\mathrm{no}.\;\mathrm{of}\;\mathrm{seeds}}\times 100$$

### 2.4. Cytogenetic Analysis in Root Tips of P. sativum L.

#### Mitotic Index (MI) Analysis

The cytotoxicity test was performed on the root tips of the germinated seedlings of seeds treated with 0.1, 0.2, 0.3, 0.4, and 0.5% of ME and IM. Roots were collected and soaked in a fixation solution (ethanol:glacial acetic acid, 3:1) for 24 h, transferred to a 70% ethanol solution, and stored at 50 °C until microscopic analysis. To prepare each slide, two roots per sample were hydrolyzed in 1 N HCl for 10 min, and the root tips were dyed with 2% acetic orcein for 10 min. The chromosome preparation from the root tips was performed as described by Qian [26] with minor modifications [27]. MI in metaphase and anaphase plates was examined using a light microscope under oil immersion (100×). A minimum of 500 cells were scored for each slide. All slides were examined blind and coded.

### 2.5. Agroclimatic Conditions of the Experimental Site

**Figure 1:**
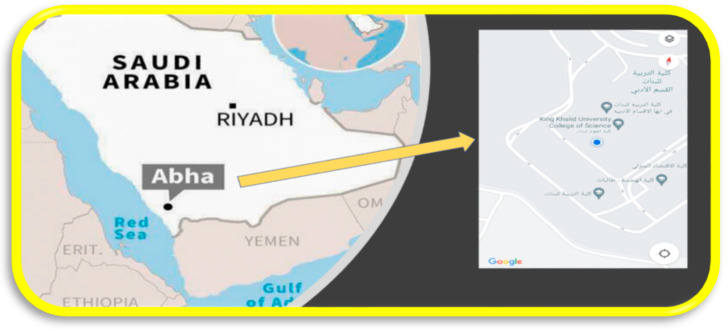
Location of College of Science, King Khalid University, Abha, K.S.A.

The experiments were conducted at the fields of the Department of Botany, Science College, Alfarra Campus, King Khalid University, Abha, Saudi Arabia from October to December (Figure 1), during which the temperature ranges from 25 to 21.9 °C. Abha is located in Aseer, in the southern part of Saudi Arabia, at a height of approximately 2270 m above sea level. Abha has a semiarid climate due to its great elevation. 

### 2.6. Collection and Fixation of Buds

Flower buds were taken from the control and plants derived from insecticide-treated (ME and IM) seeds at 34 days after seed germination. The flower buds were fixed in Carnoy’s fluid (alcohol:chloroform:acetic acid 6:3:1 ratio) for 40 min, transferred to propionic acid that was saturated with ferric acetate for a period of 24 h, and lastly stored in 70% alcohol. Anthers were squashed in 0.5% propionocarmine. Normal butanol alcohol (NBA) series were used to produce permanent slides, which were mounted in Canada balsam and dried at 45 °C. 

### 2.7. Genotoxicity Test Performed on Pollen Mother Cells (PMCs) of P. sativum L.

#### 2.7.1. Chromosomal Aberration Frequency (CAF)

Chromosomal abnormalities in metaphase and anaphase plates were examined under a light microscope with an oil immersion (100×). A minimum of 50 metaphase and anaphase plates per slide were examined for CNU, STC, BR, LG, SA, and PS.

#### 2.7.2. Micronucleus Frequency (MNF)

For the MN evaluation, from each slide, 400 cells were scored for computing the MNF. A binocular light microscope (Olympus, Japan) was used to examine the micronucleated cells (100×). The method described by Tolbert et al. [28] was used to score the micronucleus frequency. 

### 2.8. Data Analysis

Two-way ANOVA test using SPSS software (version 16.0, SPSS Inc., Chicago, IL, USA) was applied to find the significance of differences in variables. The changes were considered to be statistically significant at *p* < 0.05. All the outcomes are expressed as mean ± standard error.

## 3. Results

### 3.1. Effects of ME and IM on SG

**Figure 2:**
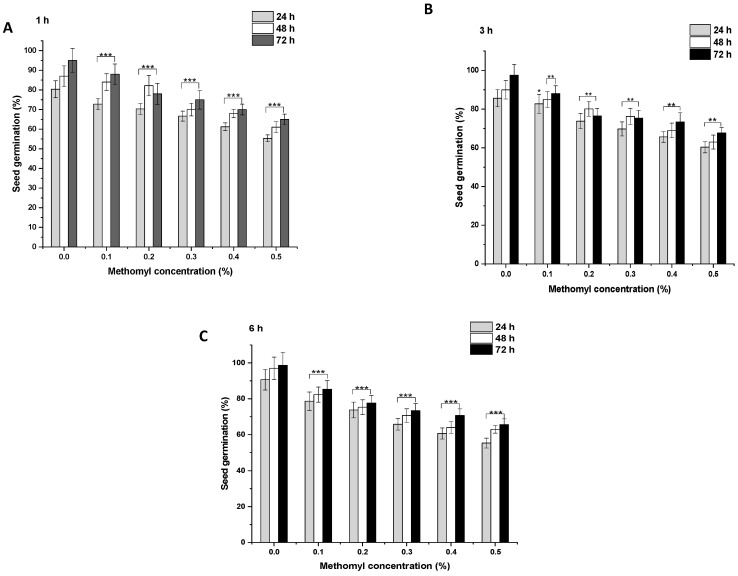
Seed germination (SG) in P. sativum seeds exposed to methomyl (ME) for (**A**) 1 h, (**B**) 3 h and (**C**) 6 h at 24, 48, and 72 h after exposure. Data represent mean and SE. The experiment was repeated thrice. * *p* < 0.05; ** *p* < 0.01 and *** *p* < 0.001 compared to control at respective time interval.

**Figure 3:**
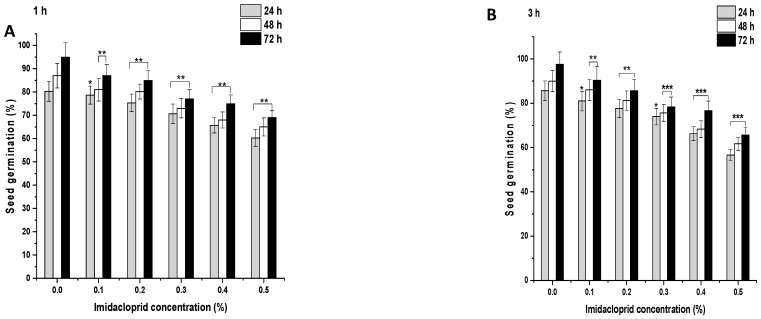

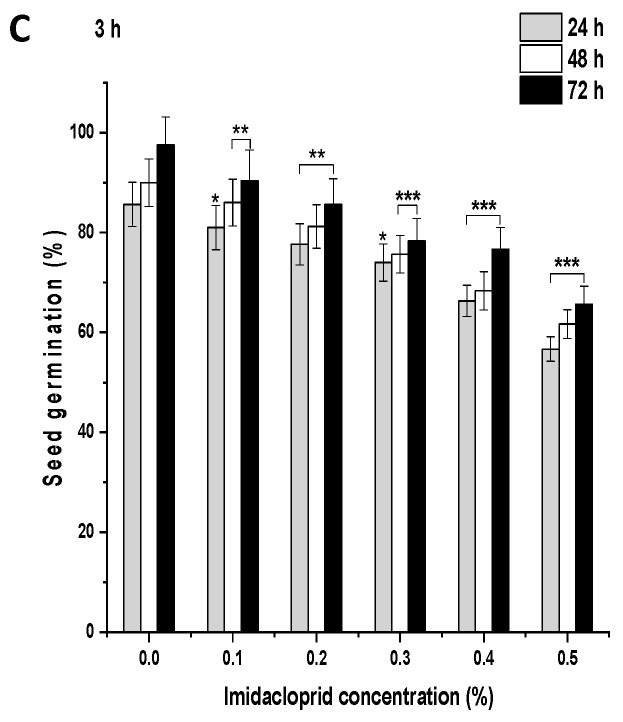
Seed germination (SG) in *P. sativum* seeds exposed to imidacloprid (IM) for (**A**) 1 h, (**B**) 3 h, and (**C**) 6 h at 24, 48, and 72 h after exposure. Data represent mean and SE. The experiment was repeated thrice. * *p* < 0.05; ** *p* < 0.01 and *** *p* < 0.001 compared to control at respective time interval.

In the control group, after 1 h, 80.33% of the seeds germinated, which increased to 87% and 95% at 48 and 72 h, respectively (Figure 2A and Figure 3A). ME and IM treatments from 0.1% to 0.5% for 1 h resulted in a significant inhibition of SG (*p* < 0.001) at 24 h in comparison to the controls. A similar trend was observed in the SG pattern at 48 and 72 h. After 1 h treatment with ME and IM, maximal SG was observed at a concentration of 0.1% at 24 h (ME: 72.77%, IM: 78.66%), at 48 h (ME: 84%, IM: 81%), and at 72 h (ME: 88%, IM: 87%). Minimal SG was reported at a 0.5% concentration at 24 h (ME: 55.33%, IM: 60.33%), at 48 h (ME: 61%, IM: 65%), and at 72 h (ME: 65%, IM: 69%). In the control group, after 3 h, 85.66% of the seeds germinated, which increased to 89.99% and 97.55% at 48 and 72 h, respectively (Figure 2B and Figure 3B). ME and IM treatments from 0.1% to 0.5% for 3 h resulted in significant inhibition of SG (*p* < 0.01 and *p* < 0.001) at 24 h compared to the control group. A similar trend in the SG pattern was observed at 48 and 72 h. The highest SG was reported after 3 h of treatment with ME and IM at a concentration of 0.1% at 24 h (ME: 82.77%, IM: 80.99%), at 48 h (ME: 85%, IM: 86%), and at 72 h (ME: 88%, IM: 90.33%). The lowest SG was observed at a 0.5% concentration at 24 h (ME: 60.33%, IM: 56.66%), at 48 h (ME: 63%, IM: 61.66%), and at 72 h (ME: 90.33%, IM: 65.66%). In the control group of 6 h, 90.66% of the seeds germinated, which increased to 96.99% and 98.66% at 48 and 72 h, respectively (Figure 2C and Figure 3C). ME and IM treatment from 0.1% to 0.5% for 6 h resulted in a significant inhibition of SG (*p* < 0.001) at 24 h in comparison to the control group. A similar SG pattern was observed at 48 and 72 h. After 6 h exposure with ME and IM, maximal SG occurred at a concentration of 0.1% at 24 h (ME: 78.66%, IM: 76.99%), at 48 h (ME: 82.33%, IM: 79%), and at 72 h (ME: 85.33%, IM: 90.33%). The lowest SG was reported at a 0.5% concentration at 24 h (ME: 55.33%, IM: 47.33%), at 48 h (ME: 63%, IM: 55.66%), and at 72 h (ME: 65.66%, IM 60.66%).

### 3.2. Effects of ME and IM on RL

In untreated seeds, the radicle length (RL) increased with an increase in time interval after treatment with double-distilled water for 1 h: 1.0 ± 0.06 at 24 h, 1.56 ± 0.07 at 48 h, and 2.98 ± 0.05 at 72 h (Table 1, Table 2 and Table 3). For 1 h, 0.1% to 0.5% ME and IM treatment resulted in a significant inhibition of RL (*p* < 0.05 and *p* < 0.01) compared to the control group. A similar trend was observed in the RL pattern at 48 h and 72 h. Maximal RL was found at a 0.1% concentration at 24 h (ME: 0.77 ± 0.4, IM: 0.69 ± 0.02), at 48 h (ME: 1.32 ± 0.08, IM: 1.45 ± 0.02), and at 72 h (ME: 2.5 ± 0.03, IM: 2.5 ± 0.06). Minimal RL after 1 h treatment with ME and IM at a concentration of 0.5% occurred at 24 h (ME: 0.42 ± 0.03, IM: 0.50 ± 0.05), at 48 h (ME: 1.00 ± 0.04, IM: 0.60 ± 0.06), and at 72 h (ME: 1.55 ± 0.45, IM: 0.90 ± 0.05). The RL of untreated seeds increased with time, reaching 1.25 ± 0.20 at 24 h, 1.98 ± 0.40 at 48 h, and 3.15 ± 0.98 at 72 h following 3 h of treatment with double-distilled water (Table 1, Table 2 and Table 3). Meanwhile, 3 h ME and IM treatment of 0.1% to 0.5% resulted in a significant inhibition of RL (*p* < 0.05 and *p* < 0.01) in comparison to the control group. A similar pattern of RL was observed at 48 and 72 h. After 3 h treatment with ME and IM, maximal RL was found at a concentration of 0.1% at 24 h (ME: 0.99 ± 0.04, IM: 0.89 ± 0.02), at 48 h (ME: 1.45 ± 0.30, IM: 1.45 ± 0.02), and at 72 h (ME: 2.50 ± 0.68, IM: 2.23 ± 0.06). Minimal RL was found at a 0.5% at 24 h (ME: 0.54 ± 0.03, IM: 0.52 ± 0.05), at 48 h (ME: 0.85 ± 0.04, IM: 0.63 ± 0.05), and at 72 h (ME: 1.00 ± 0.03, IM: 0.94 ± 0.045). After 6 h of treatment with double-distilled water, RL increased with time, reaching 1.32 ± 0.06 at 24 h, 2.92 ± 0.07 at 48 h, and 3.25 ± 0.94 at 72 h (Table 1, Table 2 and Table 3). Meanwhile, 6 h ME and IM treatments of 0.1% to 0.5% resulted in a significant inhibition of RL (*p* < 0.05 and *p* < 0.01) compared to the control group. A similar trend in the RL pattern was observed at 48 and 72 h. Maximal RL was observed at a concentration of 0.1% at 24 h (ME: 0.73 ± 0.031, IM: 0.75 ± 0.01), at 48 h (ME: 0.99 ± 0.02, IM: 0.99 ± 0.02), and at 72 h (ME: 1.45 ± 0.09, IM:1.55 ± 0.06). Minimal RL was observed at a 0.5% concentration at 24 h (ME: 0.48 ± 0.001, IM: 0.45 ± 0.05), at 48 h (ME: 0.56 ± 0.012, IM: 0.50 ± 0.05), and at 72 h (ME: 0.67 ± 0.09, IM: 0.65 ± 0.045) after 6 h treatment with ME and IM.

### 3.3. Effects of ME and IM on MI

**Figure 4:**
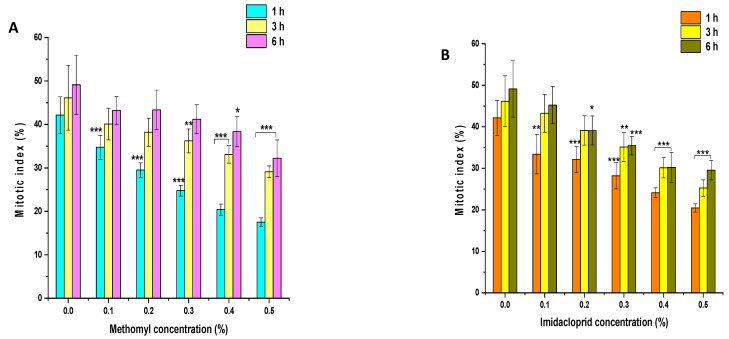
Effect of insecticide exposure on mitotic index in root tip cells of *P. sativum* seeds exposed to different concentration of (**A**) methomyl (ME) and (**B**) imidacloprid (IM) for 1, 3, and 6 h at 24 h after exposure. Data represent mean and SE. The experiment was repeated thrice. * *p* < 0.05; ** *p* < 0.01 and *** *p* < 0.001 compared to control at respective time interval.

The effects of insecticides ME and IM on the MI of *P. sativum* root tip cells are presented in Figure 4A,B. In the control group, MI values were approximately 42%, 46%, and 49% in seeds treated with double-distilled water for 1, 3, and 6 h, respectively. In comparison to the control group, a significant decline (*p* < 0.001) in MI was observed in seeds treated with 0.1% to 0.5% of ME for 1 h. ME treatment of 0.1 to 0.2% showed a nonsignificant decline (*p* > 0.05), while 0.3% to 0.5% ME treatment resulted in a significant inhibitory effect (*p* < 0.01 and *p* < 0.001) on MI in comparison to the control group following 3 h treatment. The ME treatment of 0.1 to 0.3% resulted in a nonsignificant decline (*p* > 0.05), while 0.4% to 0.5% ME treatment resulted in a significant decrease (*p* < 0.05 and *p* < 0.001) in MI in comparison to the control group following treatment for 6 h. Compared to the control group, a significant decrease (*p* < 0.001) in MI was observed in seeds treated with 0.1% to 0.5% IM for 1 h. IM treatment of 0.1 to 0.2% resulted in a nonsignificant decline (*p* > 0.05), while IM treatment of 0.3% to 0.5% showed a significant inhibitory effect (*p* < 0.01 and *p* < 0.001) on MI in comparison to the control group when treated for 3 h. The IM treatment of 0.1% resulted in a nonsignificant decline (*p* > 0.05), while the IM treatment of 0.2% to 0.5% had a significant inhibitory effect (*p* < 0.05 and *p* < 0.001) on MI compared to the control group following 6 h treatment.

### 3.4. Effects of ME and IM on CAF

**Figure 5:**
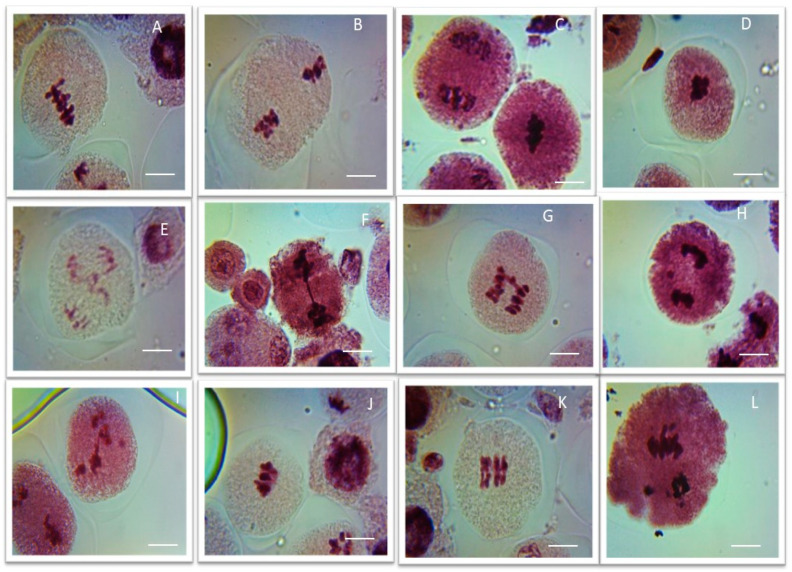
Representative images of various meiotic aberrations observed in pollen mother cells of methomyl (ME) and Imidacloprid (IM) exposed *P. sativum* seeds. (**A**,**B**) Clumped nuclei (CNU) at metaphases I and II; (**C**,**D**) stickiness (STC) at metaphases I and II; (**E**–**G**) bridges (BR) at anaphases I and II; (**H**,**I**) laggards (LG) at anaphases I and II; (**J**,**K**) secondary association (SA) at metaphases I and II; (**L**) precocious separation (PS) at anaphase II; *Scale bars = 10 μm*.

In the control group, no aberrant metaphases I and II or anaphases I and II were reported in the PMCs of *P. sativum* after treatment with double distilled water for 1, 3, and 6 h (Table 4, Table 5 and Table 6 and Figure 5). In plants treated with ME for 1 h, the percentage of aberrant metaphases I and II and anaphases I and II increased as the concentration of ME increased. The common aberrations were PS (0.52%) at a 0.1% concentration and STC (0.55%), BR (0.88%), and PS (0.58%) at a 0.2% concentration in comparison to the control group. However, a 0.3% to 0.5% increase in the concentration resulted in a very significant increase (*p* < 0.01) in the number of aberrant cells. The maximal number of aberrant cells was found at a concentration of 0.5% for CNU (2.26%), STC (2.45%), BR (1.76%), LG (1.50%), SA (1.66%), and PS (1.12%) compared to the control group. In plants treated with ME for 3 h, the percentage of aberrant metaphases I and II and anaphases I and II increased as the concentration of ME increased. At a 0.1% concentration, common aberrations were CNU (1.05%), LG (1.55%), SA (1.26%), and PS (1.26%); at a 0.2% concentration, they were CNU (2.56%), BR (1.48%) LG (2.20%), STC (1.14%), and PS (2.67%) in comparison to the control group. Further, a 0.3% to 0.5% increase in the concentration resulted in a very significant increase (*p* < 0.01) in the number of aberrant cells. The maximal number of aberrant cells was found at a concentration of 0.5% in CNU (4.12%), STC (3.0%), BR (4.75%), LG (5.40%), SA (6.76%), and PS (6.22%) compared to the control group.

In plants treated with ME for 6 h, the percentage of aberrant metaphases I and II and anaphases I and II increased with an increase in the concentration of ME. At a 0.1% concentration, common aberrations were CNU (1.56%), LG (0.81%), SA (3.56%), and PS (1.67%) in comparison to the control group. Meanwhile, a 0.2% to 0.5% increase in the concentration resulted in a very significant increase (*p* < 0.01) in the number of aberrant cells. The maximal number of aberrant cells was found at a concentration of 0.5%, namely, CNU (9.76%), STC (9.51%), BR (12.11%), LG (12.11%), SA (13.05%), and PS (10.44%), compared to the control group.

In plants treated with IM for 1 h, the percentage of aberrant metaphases I and II and anaphases I and II increased as the concentration of IM increased. At the lowest concentration (0.1%), no chromosomal aberrations were found; at 0.2%, aberrations were CNU (0.67%), BR (0.77%), LG (0.98%), and PS (0.80%) in comparison to the control group. A further increase in the IM concentration from 0.3% to 0.5% resulted in a very significant increase (*p* < 0.01) in the number of aberrant cells. The maximal number of aberrant cells was found at a concentration of 0.5%, namely, CNU (2.23%), STC (1.94%), BR (2.16%), LG (2.1%), SA (2.1%), and PS (2.3%), compared to the control.

Abbreviations used for Table 4, Table 5 and Table 6: clumped nuclei (CNU), stickiness (STC), bridges (BR), laggards (LG), secondary association (SA), and precocious separation (PS).

In plants treated with IM for 3 h, the percentage of aberrant metaphases I and II, and anaphases I and II increased with an increase in the concentration of IM. At the lowest concentration (0.1%), chromosomal aberrations included CNU (1.72%), BR (1.05%), LG (1.23%), and PS (1.24%) compared to the control. Further increases in the IM concentration from 0.2% to 0.5% resulted in an increase (*p* < 0.05 and *p* < 0.01) in the number of aberrant cells. The maximal number of aberrant cells was found at a concentration of 0.5%, namely, CNU (5.13%), STC (6.12%), BR (5.63%), LG (6.29%), SA (4.80%), and PS (4.20%), in comparison to the control group. In plants treated with IM for 6 h, the percentage of aberrant metaphases I and II, and anaphases I and II increased with an increase in the IM concentration. The percentage of aberrant cells increased with an increase in the concentration of IM (0.1 to 0.5%) compared to the control. The maximal number of aberrant cells was found at a concentration of 0.5%, namely, CNU (8.7%), STC (6.8%), BR (15.55%), LG (12.50%), SA (11.9%), and PS (13.21%), in comparison to the control group.

### 3.5. Effects of ME and IM on MNF

**Figure 6:**
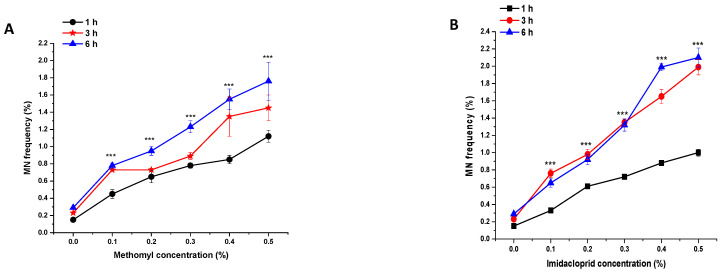
Micronucleus frequency in pollen-mother cells of *P. sativum* seeds treated with different concentrations of (**A**) methomyl (ME) and (**B**) imidacloprid (IM) for 1, 3, and 6 h. Data are means of three replicates ± SE. *** *p* < 0.001 compared to control at respective time interval.

**Figure 7:**
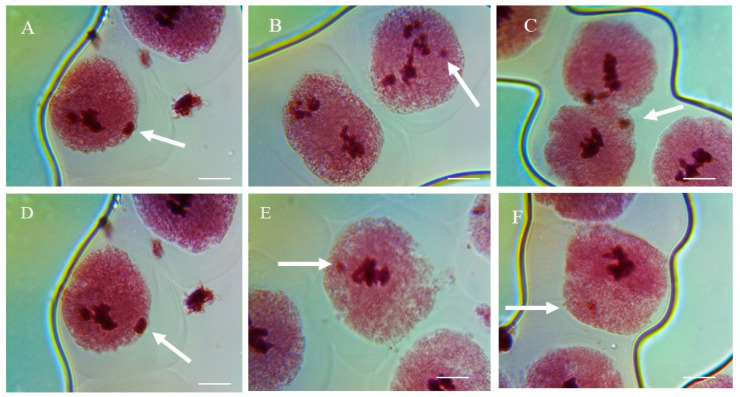
Representative images of micronucleated cells (**A**–**F**) in pollen-mother cells of *P. sativum* seeds treated with different concentrations of methomyl (ME) and imidacloprid (IM) for 1, 3, and 6 h. Arrows indicate micronuclei. *Scale bars = 10 μm*.

In the control group, the MNF was approximately 0.15%, 0.23%, and 0.29% in the PMCs of *P. sativum* treated with double-distilled water for 1, 3, and 6 h, respectively (Figure 6 and Figure 7). Compared to the control group, a highly significant increase (*p* < 0.001) in MNF was observed in plants treated with 0.1% to 0.5% of ME and IM for 1, 3, and 6 h. In treated plants, the maximal MNF was reported at a concentration of 0.5% at 1 h with ME (1.12%), at 3 h with IM (1.19%), and at 6 h with IM (2.10%). The minimal MNF was reported at a concentration of 0.1% at 1 h with IM (0.33%), at 3 h with ME (0.73%), and at 6 h with IM (0.65%).

## 4. Discussion

The findings of this study reveal that the exposure of seeds to higher doses of ME and IM not only delays but also prevents the germination of *P. sativum* seeds. The germination potential of seeds is very sensitive to environmental factors. Studies showed that the exposure of seeds to heavy metals and various mutagenic agents significantly reduces seed germination [29,30,31]. Higher concentrations of insecticides kitazin and endosulfan prevented germination in brinjal plants (*Solanum melongena* L.) [32], *Solanum lycopersicum*, *Capsicum annuum*, *Solanum melongena*, Zea mays, *P. sativum*, *Typha latofolia*, and *Brassica nigra* [33,34]. The presence of pesticides in soil can hinder the uptake of vital nutrients by plant roots, resulting in nutrient deprivation and growth retardation [35]. The lengthening of radicles is associated with cell multiplication. However, ME and IM exhibited an inhibitory effect on proliferation of cells in this study, as shown by mitotic index results. This could have been caused by changes in the expression of specific genes regulating the cell cycle. 

In previous research, the mutagenic action of ME and IM was demonstrated in *P. sativum* and *Allium cepa* [36,37]. In our study, we observed a similar effect of ME and IM on *P. sativum* seeds. In root tips grown from *P*. *sativum* seeds, ME and IM demonstrated a significant inhibitory effect on mitosis, which may have been due to the repressing effect on spindle fibers [38,39], protein synthesis, RNA, and DNA [40,41,42]. Further, by preventing CDK1/cyclin activation, glyphosate stops the cell cycle at the G2-M stage [34,43]. Previous investigations also revealed analogous effects of organophosphates on biological systems [44,45,46].

Micronucleus frequency induction was reported in *P. sativum* PMCs. The formation of MNF indicated a mutagenic action caused by damage, no repair, and misreporting in parental cells [47] caused by ME and IM. Other investigations found similar genotoxic effects of insecticides on *A. sativum* [48] and *V. faba* [49].

Plant cytological abnormalities can be used as a reliable marker for detecting environmental contaminants that may pose grave genetic risks. Following ME and IM treatment, several kinds of CAF were found in *P. sativum*, including CNU, STC, BR, LG, SA, and PS. These findings suggest that these chemicals have the potential to cause meiotic abnormalities, as reported by previous researchers [50,51,52]. Specifically, these insecticides may cause chromosomal anomalies by inhibiting spindle proteins and inducing sister chromatid exchange [33,53]. Free radicals cause genomic instability in cells. Reactive oxygen species are very unstable and can disrupt the cytoskeleton, induce energy metabolism imbalance, and damage DNA, resulting in chromosomal abnormalities [37,54]. DNA damage is an initial biological phenomenon that can impair biological structures and processes, and cause genotoxic syndromes that are associated with the development of carcinogenic processes [21,22,23]. Several factors, including DNA damage caused by insecticides, promote carcinogenic developments in a broad range of species, as per a recent analysis [24].

The genotoxic effect of ME and IM found in this study could have partly been due to the oxidative stress caused by these agents. Several studies showed that these chemicals change the redox status of plant cells, which supports this theory [54,55]. Under the experimental settings used in this study, ME and IM had a potent genotoxic effect on the *P. sativum* plant. More research on the quality of products obtained from seeds/plants exposed to these chemicals is needed in terms of disease susceptibility, nutritional value, and susceptibility to acclimated stress.

## 5. Conclusions

The findings of the current study suggest that insecticides can have a genotoxic effect on nontarget organisms such as plants. Both insecticides at higher doses had harmful effects, as evident from the high incidence of SG, RL, MI, CAF, and MNF in the *P. sativum* model plant used in the study. Insecticide dealers frequently advise farmers to use insecticides in amounts that are double the recommended dose, which can have detrimental cytogenetic effects and inhibit plant growth. Therefore, the use of insecticide in excess of the recommended dose should be avoided. Farmers and insecticide sellers must be educated on the suitable and optimal application of insecticides. The impact of these insecticides on nontarget host plants should be explored further at the gene expression level in order to determine the means by which they have adverse effects on nontarget plants.

## Figures and Tables

**Table 1:** Effect of ME and IM on RL of *P. sativum* at different concentrations for 1 h.

CN (%)	RL (cm)		
	24 h	48 h	72 h
0.0	1.0 ± 0.06	1.56 ± 0.07	2.98 ± 0.05
ME			
0.1	0.77 ± 0.04 *	1.32 ± 0.08	2.50 ± 0.03 *
0.2	0.61 ± 0.03 **	1.15 ± 0.31 **	2.15 ± 0.34 **
0.3	0.63 ± 0.08 ***	0.99 ± 0.04 **	1.72 ± 0.44 **
0.4	0.52 ± 0.02 ***	1.12 ± 0.50 **	1.65 ± 0.33 **
0.5	0.42 ± 0.03 ***	1.00 ± 0.04 **	1.55 ± 0.45 **
IM			
0.1	0.69 ± 0.02 *	1.45 ± 0.02	2.50 ± 0.06 *
0.2	0.64 ± 0.04 **	1.25 ± 0.10 *	2.25 ± 0.90 **
0.3	0.55 ± 0.03 **	0.99 ± 0.05 **	1.88 ± 0.12 **
0.4	0.59 ± 0.06 **	0.97 ± 0.03 **	0.99 ± 0.02 ***
0.5	0.50 ± 0.05 **	0.60 ± 0.06 *	0.90 ± 0.05 ***

* *p* < 0.05; ** *p* < 0.01; *** *p* < 0.001 compared to control group; data are means of three replicates ± SD; 0.0 = control group, CN = concentration.

**Table 2:** Effect of ME and IM on radical length of *P. sativum* at different concentrations for 3 h.

CN (%)	RL (cm)		
	24 h	48 h	72 h
0.0	1.25 ± 0.20	1.98 ± 0.40	3.15 ± 0.98
ME			
0.1	0.99 ± 0.04 *	1.45 ± 0.30	2.50 ± 0.68 *
0.2	0.80 ± 0.02 ***	1.32 ± 0.31 **	1.78 ± 0.68 **
0.3	0.73 ± 0.04 **	1.00 ± 0.012 **	1.55 ± 0.44 **
0.4	0.62 ± 0.02 **	0.99 ± 0.03 **	1.25 ± 0.05 **
0.5	0.54 ± 0.03 **	0.85 ± 0.04 **	1.00 ± 0.03 **
IM			
0.1	0.89 ± 0.02 *	1.45 ± 0.02 **	2.23 ± 0.06 **
0.2	0.74 ± 0.04 **	1.35 ± 0.08 **	2.12 ± 0.23 **
0.3	0.65 ± 0.03 **	1.22 ± 0.05 **	1.98 ± 0.12 **
0.4	0.61 ± 0.04 **	0.99 ± 0.03 **	1.00 ± 0.021 **
0.5	0.52 ± 0.05 **	0.63 ± 0.05 **	0.94 ± 0.045 **

* *p* < 0.05; ** *p* < 0.01; *** *p* < 0.001 compared to control group; data are means of three replicates ± SDs; 0.0 = control group, CN = concentration.

**Table 3:** Effect of ME and IM on RL of *P. sativum* at different concentrations for 6 h.

CN (%)	RL (cm)		
	24 h	48 h	72 h
0.0	1.32 ± 0.060	2.92 ± 0.070	3.25 ± 0.94
ME			
0.1	0.73 ± 0.031 **	0.99 ± 0.020 **	1.45 ± 0.09 *
0.2	0.70 ± 0.230 **	0.76 ± 0.040 **	1.12 ± 0.04 **
0.3	0.64 ± 0.42 0 ***	0.73 ± 0.040 **	0.99 ± 0.07 **
0.4	0.54 ± 0.012 **	0.64 ± 0.030 **	0.89 ± 0.09 **
0.5	0.48 ± 0.001 *	0.56 ± 0.012 **	0.67 ± 0.09 **
IM			
0.1	0.75 ± 0.01 **	0.99 ± 0.02 **	1.55 ± 0.06 0 **
0.2	0.73 ± 0.04 ***	0.79 ± 0.08 ***	1.12 ± 0.230 ***
0.3	0.62 ± 0.03 ***	0.70 ± 0.05 ***	0.99 ± 0.120 ***
0.4	0.50 ± 0.04 ***	0.65 ± 0.03 ***	0.75. ± 0.021 ***
0.5	0.45 ± 0.05 ***	0.50 ± 0.05 ***	0.65 ± 0.045 ***

* *p* < 0.05; ** *p* < 0.01; *** *p* < 0.001 compared to control group; data are means of three replicates ± SD; 0.0 = control group, CN = concentration.

**Table 4:** Percentage of meiotic abnormality in metaphases I and II and anaphases I and II of plates of *P. sativum* in PMCS exposed to different concentrations of ME and IM for 1 h.

CN (%)	CNU	STC	BR	LG	SA	PS
0.0	0.00 ± 0.00	0.00 ± 0.00	0.00 ± 0.00	0.00 ± 0.00	0.00 ± 0.00	0.00 ± 0.00
ME						
0.1	0.00 ± 0.00	0.00 ± 0.00	0.00 ± 0.00	0.00 ± 0.00	0.00 ± 0.00	0.52 ± 0.033 ^b^
0.2	0.00 ± 0.000	0.55 ± 0.003 ^b^	0.88 ± 0.012 ^b^	0.00 ± 0.000	0.00 ± 0.000	0.58 ± 0.035 ^b^
0.3	0.88 ± 0.005 ^b^	0.87 ± 0.005 ^b^	1.43 ± 0.041 ^b^	0.65 ± 0.023 ^b^	0.66 ± 0.006 ^b^	0.73 ± 0.037 ^b^
0.4	1.17 ± 0.039 ^b^	1.19 ± 0.060 ^b^	1.56 ± 0.045 ^b^	1.51 ± 0.043 ^b^	0.99 ± 0.003 ^b^	0.82 ± 0.057 ^b^
0.5	2.26 ± 0.033 ^b^	2.45 ± 0.034 ^b^	1.76 ± 0.036 ^b^	1.50 ± 0.057 ^b^	1.66 ± 0.088 ^b^	1.12 ± 0.063 ^b^
IM						
0.1	0.00 ± 0.00	0.00 ± 0.00	0.00 ± 0.00	0.00 ± 0.00	0.00 ± 0.00	0.00 ± 0.00
0.2	0.67 ± 0.057 ^b^	0.00 ± 0.000	0.77 ± 0.010 ^b^	0.98 ± 0.03 ^ab^	0.00 ± 0.000	0.80 ± 0.057 ^b^
0.3	1.23 ± 0.033 ^bc^	0.92 ± 0.035 ^b^	0.98 ± 0.005 ^b^	1.59 ± 0.43 ^b^	0.63 ± 0.033 ^b^	1.37 ± 0.021 ^b^
0.4	1.85 ± 0.050 ^b^	1.63 ± 0.06 ^bc^	1.27 ± 0.033 ^b^	1.93 ± 0.32 ^b^	1.69 ± 0.030 ^b^	2.12 ± 0.430 ^b^
0.5	2.23 ± 0.033 ^b^	1.94 ± 0.032 ^b^	2.16 ± 0.006 ^b^	2.10 ± 0.57 ^b^	2.10 ± 0.210 ^b^	2.31 ± 0.22 ^b^

^a^*p* < 0.001; ^b^ *p* < 0.01; ^c^ *p* < 0.05 compared to control group. Data are means of three replicates ±SE. 0.0 = control group, CN = concentration.

**Table 5:** Percentage of meiotic abnormality in metaphases I and II and anaphases I and II of plates of *P. sativum* in PMCS exposed to different concentrations of ME and IM for 3 h.

CN (%)	CNU	STC	BR	LG	SA	PS
0.0	0.00 ± 0.00	0.00 ± 0.00	0.00 ± 0.00	0.00 ± 0.00	0.00 ± 0.00	0.00 ± 0.00
ME						
0.1	1.05 ± 0.012 ^b^	0.00 ± 0.00	0.00 ± 0.00	1.55 ± 0.060 ^a^	1.26 ± 0.033 ^b^	1.26 ± 0.62
0.2	2.56 ± 0.033 ^b^	0.00 ± 0.00	1.48 ± 0.31 ^b^	2.20 ± 0.057 ^b^	2.14 ± 0.91 ^b^	2.67 ± 0.73
0.3	3.60 ± 0.057 ^b^	0.00 ± 0.00	1.76 ± 0.51 ^b^	3.33 ± 0.91	3.33 ± 0.81 ^b^	3.10 ± 0.81
0.4	3.59 ± 0.091 ^b^	1.85 ± 0.25 ^b^	3.22 ± 1.22 ^b^	4.30 ± 1.20	5.36 ± 1.22 ^b^	4.11 ± 0.75
0.5	4.12 ± 0.219 ^b^	3.00 ± 0.98 ^b^	4.75 ± 1.78 ^b^	5.40 ± 1.50	6.76 ± 1.57	6.22 ± 1.91
IM						
0.1	1.72 ± 0.032 ^b^	0.00 ± 0.000	1.05 ± 0.03 ^b^	1.23 ± 0.040 ^b^	0.00 ± 0.000	1.24 ± 0.21 ^b^
0.2	1.72 ± 0.021 ^b^	1.26 ± 0.031	2.14 ± 0.013 ^b^	2.20 ± 0.057 ^b^	1.30 ± 0.057 ^b^	1.86 ± 0.33 ^b^
0.3	2.32 ± 0.062 ^b^	2.56 ± 0.21 ^b^	3.28 ± 0.76 ^b^	3.80 ± 0.91 ^b^	2.73 ± 0.033 ^b^	2.15 ± 0.43 ^bc^
0.4	3.55 ± 0.057 ^b^	3.08 ± 0.0.42 ^b^	4.29 ± 0.94 ^b^	4.90 ± 1.15 ^b^	3.15 ± 0.99 ^b^	3.19 ± 0.95 ^bc^
0.5	5.13 ± 0.95 ^b^	6.12 ± 1.12 ^bc^	5.63 ± 1.25 ^b^	6.29 ± 1.72 ^b^	4.80 ± 1.22 ^bc^	4.20 ± 1.23 ^bc^

^a^*p* < 0.001; ^b^ *p* < 0.01; ^c^ *p* < 0.05 compared to control group. Data are means of three replicates ±SE. 0.0 = control group, CN = concentration.

**Table 6:** Percentage of meiotic abnormality in metaphases I and II and anaphases I and II of plates of *P. sativum* in PMCS exposed to different concentrations of ME and IM for 6 h.

CN (%)	CNU	STC	BR	LG	SA	PS
0.0	0.00 ± 0.00	0.00 ± 0.00	0.00 ± 0.00	0.00 ± 0.00	0.00 ± 0.00	0.00 ± 0.00
ME						
0.1	1.05 ± 0.012 ^b^	0.00 ± 0.00	0.00 ± 0.00	1.55 ± 0.060 ^a^	1.26 ± 0.033 ^b^	1.26 ± 0.62
0.2	2.56 ± 0.033 ^b^	0.00 ± 0.00	1.48 ± 0.31 ^b^	2.20 ± 0.057 ^b^	2.14 ± 0.91 ^b^	2.67 ± 0.73
0.3	3.60 ± 0.057 ^b^	0.00 ± 0.00	1.76 ± 0.51 ^b^	3.33 ±0.91	3.33 ± 0.81 ^b^	3.10 ± 0.81
0.4	3.59 ± 0.091 ^b^	1.85 ± 0.25 ^b^	3.22 ± 1.22 ^b^	4.30 ± 1.20	5.36 ± 1.22 ^b^	4.11 ± 0.75
0.5	4.12 ± 0.219 ^b^	3.00 ± 0.98 ^b^	4.75 ± 1.78 ^b^	5.40 ± 1.50	6.76 ± 1.57	6.22 ± 1.91
IM						
0.1	1.72 ± 0.032 ^b^	0.00 ± 0.000	1.05 ± 0.03 ^b^	1.23 ± 0.040 ^b^	0.00 ± 0.000	1.24 ± 0.21 ^b^
0.2	1.72 ± 0.021 ^b^	1.26 ± 0.031	2.14 ± 0.013 ^b^	2.20 ± 0.057 ^b^	1.30 ± 0.057 ^b^	1.86 ± 0.33 ^b^
0.3	2.32 ± 0.062 ^b^	2.56 ± 0.21 ^b^	3.28 ± 0.76 ^b^	3.80 ± 0.91 ^b^	2.73 ± 0.033 ^b^	2.15 ± 0.43 ^bc^
0.4	3.55 ± 0.057 ^b^	3.08 ± 0.0.42 ^b^	4.29 ± 0.94 ^b^	4.90 ± 1.15 ^b^	3.15 ± 0.99 ^b^	3.19 ± 0.95 ^bc^
0.5	5.13 ± 0.95 ^b^	6.12 ± 1.12 ^bc^	5.63 ± 1.25 ^b^	6.29 ± 1.72 ^b^	4.80 ± 1.22 ^bc^	4.20 ± 1.23 ^bc^

^a^*p* < 0.001; ^b^ *p* < 0.01; ^c^ *p* < 0.05 compared to control group. Data are means of three replicates ± SE. 0.0 = control group, CN= concentration.

## Data Availability

Not applicable.
